# An Experimental Investigation on the Thermo-Rheological Behaviors of Lactic Acid-Based Natural Deep Eutectic Solvents

**DOI:** 10.3390/ma15114027

**Published:** 2022-06-06

**Authors:** Yousef Elhamarnah, Mashael AlRasheedi, Wadha AlMarri, Asma AlBadr, Alanoud AlMalki, Nora Mohamed, Izzah Fatima, Mustafa Nasser, Hazim Qiblawey

**Affiliations:** 1 Department of Chemical Engineering, College of Engineering, Qatar University, Doha P.O. Box 2713, Qatar; ye1206348@student.qu.edu.qa (Y.E.); ms1702133@student.qu.edu.qa (M.A.); wa1708179@student.qu.edu.qa (W.A.); aa1601691@student.qu.edu.qa (A.A.); aa1702304@student.qu.edu.qa (A.A.); ns1701580@qu.edu.qa (N.M.); if1703890@student.qu.edu.qa (I.F.); hazim@qu.edu.qa (H.Q.); 2 Gas Processing Center, College of Engineering, Qatar University, Doha P.O. Box 2713, Qatar

**Keywords:** β-alanine, rheology, choline chloride, viscoelastic, betaine, natural deep eutectic solvents, lactic acid

## Abstract

The rheological studies of Lactic Acid (LA)-based Natural Deep Eutectic Solvents (NADES) are provided in the present investigation. Those mechanisms were also studied in which three distinct Hydrogen Bond Acceptors (HBAs) of Choline Chloride (ChCl), Betaine (Be), and β-Alanine (β-Al), after being added to a specific Hydrogen Bond Donor (HBD) at a predefined mole-to-mole ratio of 1:1, affected the rheological properties of the prepared NADES. The alterations in the rheology-related characteristics in association with the mechanical and physical properties indicate the tolerance of the material under various operational conditions in the field and show their potential utilization as environmentally suitable and feasible solvents for industrial applications. In the present research, the viscoelastic properties of the three samples of NADES were assessed along with their shear flow properties. The backward and forward temperature change in the Apparent Viscosity (AV) pattern related to the NADES system was described by a rheogram. Furthermore, the density was determined and compared with the AV while considering the temperature-related factor. On a further note, the viscoelastic characteristics were utilized in describing and investigating the network disturbance on the level of the microstructure of NADES upon frequency sweep. A series of experiments were carried out using Thermogravimetry Analysis (TGA) to investigate the thermo-physical properties to optimize them. The rheological properties of shear flow measurements were analyzed using the Bingham model that is best suited for the AV developed with the shear rate with the dynamic yield stress of three systems. The Bingham model was used to determine the lowest stress necessary to disturb the network structure and commence the flow of LA-based NADES. Overall, the viscoelastic behavior of the LA-based NADES revealed the dissimilarity between their strength and viscosity. In addition, shear flow investigations demonstrated that LA-based NADES systems exhibit non-Newtonian properties and substantial shear-thinning effects equivalent to those of alternative IL sorbents. Assessing the rheological properties of LA-based NADES is crucial for a better understanding the key challenges associated with high viscosity. Defining the transport yield stress requirements for NADES systems under different conditions benefits their future development and potentially opens the door to more challenging applications.

## 1. Introduction

The motivation for applying rheological analysis stems from the notion that the multiple qualities of materials seen in commercial applications might be linked to certain readily researched rheological properties. Numerous uses of rheology include the following: (1) rheological study of the material may provide some parameters that aid in interpreting the material’s physical or chemical structure, (2) rheological research may be used to compare comparable materials but cannot determine if a material is favorable or poor, (3) the obtained rheological characteristics can be utilized for modeling the dynamic behavior and flow of materials, and (4) studying unique rheological phenomena such as the solid–liquid transition, the Weissenberg phenomenon, and others that may be significant in certain industrial applications [1,2].

A critical component of developing sustainable systems is access to innovative and unique chemical solvents [3,4] that enable fine-tuning of their physicochemical characteristics for specific applications through chemical or physical parameter changes/variations. Recently, it was accepted that recent development in the class of green solvents complied with the majority of the disciplines of green chemistry, usually referred to as NADES [5,6,7]. These solvent classes have recently received considerable interest due to their major benefits over conventional organic solvents, Ionic Liquids (ILs), and Deep Eutectic Solvents (DESs) [8,9,10]. NADES are comprised of two components (HBA and HBD) derived from primary metabolites (sugars, alcohols, organic acids, and amines) that are plentiful in nature and far less expensive than any commercial solvent [6,11,12,13,14]. Additionally, NADES have shown significant environmental benefits over conventional solvents; they are biodegradable, sustainable, have a low toxicity, and have high thermal stability [13,15,16,17].

Rheology is an effective technique for determining the effect of many physical and chemical factors on the behavior of complex systems [18]. Additionally, rheology aids in determining the appropriateness of materials before their manufacturing and use on a wider scale. The primary goal of this work was to offer a concise summary of the present state of the art in terms of the rheological characterization of lactic acid-based NADES. As a result, we consider that this research gives critical information on how to fine-tune the HBA component in NADES (rheological characteristics) to construct sustainable systems for a variety of applications effectively. However, there are few studies demonstrating the shear flow characteristics of NADES under these circumstances, making it difficult to estimate their flow properties [9]. Additionally, the dearth of theoretical research on the viscosities of DESs has complicated classifying distinct solvents for their proper use.

Rheological measurements have been used extensively to examine the relaxation dynamics and structure–property correlations of NADESs [9]. The viscosity analysis as a function of temperature and shear rate permits the investigation of the interactions and rotations between HBA and HBD in NADES [19]. Dynamic and steady rheological techniques are also beneficial for evaluating the quality of NADES as solvents for complex operations such as liquid material pumping in a variety of industries. Oscillatory analysis has also been shown to be an effective method for determining the elastic characteristics of NADESs, which may indicate whether the solvents behave more like viscous liquids or solids, a critical parameter for pumping and processing such structured solvents. Additionally, rheology investigations have shed light on the influence of molar ratios on NADES viscosity behaviors as a function of temperature. Altamash, et al. [20] discovered that the molar ratio becomes an independent component in viscosity at higher temperatures but has a considerable effect at low temperatures. This conclusion will have a substantial impact on a variety of applications that demand low viscosity at high temperatures to decrease pumping costs and on practical solvent uses that require increased mass transfer rates [21]. When a NADES system is used in a commercial application, adding water as a co-solvent can be seen as an impurity, changing the NADES and their rheological properties. Das, et al. [22] investigated the shear flow characteristics of several hydrated and unhydrated choline chloride NADES systems for the extraction of κ-carrageenan. The flow behavior results indicated that hydrated NADES exhibited a considerably better degree of flowability and less stiff behavior, indicating that NADES may be employed in applications that need a certain amount of water, such as pumping. As a result, hydrated NADES were preferred over non-hydrated NADES due to their improved quality and extraction efficiency.

NADESs are gaining traction, as seen by the recent number of issued patents, notably in industrial activities [23]. The technological adaptability of NADESs makes them the most suitable candidates for different applications, such as their utilization in various field operations, including materials science, separation operations, chemical engineering, and several other processes related to energy [24,25,26,27,28,29]. Rheological characterization complements other approaches in explaining physical and structural changes in the NADES, such as phase separation, gelation, melting, viscoelasticity, and mechanical strength [8]. The viscoelastic examination of NADES permits the avoidance of network degradation and the determination of the elastic viscoelastic characteristics of the solvents. Three different kinds of oscillatory tests may be used to determine the rheological characteristics of systems based on NADES: (1) frequency sweep tests in which the storage modulus (*G′*) and loss modulus (*G″*) are determined as a function of frequency (ω) at a constant temperature and shear stress or strain, respectively, according to the linear viscoelastic area established in a stress/strain sweep test; (2) temperature ramp tests (or time cure tests), in which *G′* and *G″* are calculated as a function of temperature while holding the temperature constant and applying a stress/strain within the linear viscoelastic zone; and (3) time sweep tests are used to determine *G′* and *G″* as a function of time, temperature, and stress/strain.

In this regard, the research was conducted utilizing a group of distinct NADES based on the fixation of lactic acid (LA) as an HBD with three distinctive HBAs, including β-alanine (β-Al), Betaine (Be), and choline chloride (ChCl) at a definite mole-to-mole ratio of 1:1. For the determination of the actual technological worth of the generated NADES as useful solvents, closely related thermo-physio rheological characteristics, such as viscoelastic properties and steady-shear-flow properties, were studied, by considering their yield stress and apparent viscosity (AV) under various conditions, including the temperature, shear rate, and angular frequency (ω). Eventually, based on our prior studies in which LA-based NADES were combined with HBAs [20,30], it can be established that the rheology-related properties of these systems showed excellent outcomes for several critical key factors for their effective processing, equipment, and process design and for modulation of their characteristics for the eventual application. Nonetheless, to the best of our knowledge, no studies were conducted on the thermo-rheological properties of LA-based NADES [9].

## 2. Materials & Methods

### 2.1. Materials

The choline chloride has a melting point of 302 °C and a purity of 97.0% (bought from Iolitech) where β-alanine has a melting point of 258 °C and a purity ≥98%, and betaine has a melting point of 310 °C, purity ≥98%. DL-lactic acid with 85% purity and a melting point of 17 °C was acquired from Sigma Aldrich (St. Louis, MO, USA). All chemicals were used without further purification or treatment. However, the solid choline chloride in the solid state was dried under vacuum at 60 °C for about two days using a desiccator to avoid any humidity absorption. The prepared NADES were stored in sealed containers of 40 mL. The components for this set of NADES systems are summarized in Table 1.

### 2.2. NADES Preparation

The three LA-based NADES systems were prepared by mixing the HBA and HBD at a predefined mole-to-mole ratio of 1:1. The basis of selecting this molar ratio was our previous study that looked at the effect of different molar ratios of a NADES on viscosity [20]. The HBD ratio had an important impact on viscosity. The viscosity was found to increase as the molar ratio of HBA:HBD increased. Hence, at higher molar ratios, the NADES formed was not liquid at room temperature. The formed solution at molar ratios higher than 1:1 voids the concept and definition of NADES. In this study, machinery mixing (i.e., rotatory evaporator from Heidolph, Schwabach, Germany was used. The glassware was dried through the vacuum oven to ensure no traces of moisture. A KERN 770 Electronic analytical balance (Kern & Sohn, Germany), was used to weigh the raw material. Ederol filter paper was used for weighting the samples prior to their addition to the round bottom flask. A rotatory mixer was used to mix the samples using oil as a heating media. The heating took place at 60 °C and under 1 atm and at a rotational speed of 90 rpm. The samples were left to mix and heat for approximately 1 h. A transparent liquid was formed, which indicates the end of the preparation process. The resulted NADES were noticed to represent a viscous-like material [31]. The moisture contents were measured for the prepared NADES using the Karl Fischer moisture titrator and are reported in Table 2.

### 2.3. Rheological Measurements

The rheological characterizations were carried out using a strain instrument (Anton Paar Rheometer Model MCR 302). Prior to sampling the NADES to the rheometer, the NADES samples were heated and stirred on a hot plate for 10–15 min using a magnetic stirrer-heater to avoid any damage or sticky patches on the sensitive measuring plates. Meanwhile, the PC, rheometer, and air compressor were turned on while the sample heats up. Anton Paar’s RheoPlus software is in charge of controlling the rheometer. Depending on the kind of experiment and the solvent being examined, an algorithm is devised. The shear rate interval, temperature, and shear strain must all be established before the experiment can begin. The gap size was 0.104 mm; the cone was 50 mm in diameter; and the plate measurement geometry was used. The cone and plate (CP) design was selected over the parallel plate (PP) layout for this set of investigations due to the presence of tiny particles in the NADES samples (particle size 5 m).

The software accomplished the initial tasks before sampling, such as evaluating the motor efficiency, measuring the gap, and adjusting the temperature. Because of their higher viscosity, the Be:LA- and β-Al:LA-based NADES were applied to the measuring apparatus using a spatula, while the ChCl:LA was applied with a pipette. The samples were allowed to settle on the measuring plate before taking any measurements.

## 3. Experimental Methodology

### 3.1. Shear Flow Measurements

The AV is a shear flow measurement property used to examine the flow behavior and understand different values of shear rate through a rheogram. The AV for the different NADESs was determined as a function of the shear rate at different temperatures ranging from 25 to 85 °C. A freshly prepared sample was used for every temperature to prevent structural damage to the samples. Meanwhile, the shear rate versus shear stress for every sample was determined. On a further note, the viscosity was also determined as a function of time for every sample. This part of the research was separated into four separate intervals of time. During the first interval, the samples were measured for about one minute, while during the second interval, third interval, and fourth interval of time, the samples were measured for the duration of five minutes, 10 min, and 15 min, respectively. A similar process was used for all the temperatures ranging from 25 to 85 °C. Controlled strain (γ ~ 1%) experiments were measured from 0.01–1000 s^−1^. The setpoints chosen and selected on the device for the conducted experiments on the LA-based NADES were summarized in Table S1.

The AV was measured individually for each NADES as a function of the shear rate at varied temperatures from 25 to 85 °C. A fresh sample was placed at each temperature measurement to avoid structural breakdown of the sample. Analogously, the shear rate against shear stress and the apparent viscosity was determined as a function of time for the LA-NADES samples. Four different time intervals were planned for this test; the first interval measured the rheological thixotropic properties in the first minute, while the 2nd, 3rd, and 4th rounds measured the run for 5, 10, and 15 min, respectively, at a predefined temperature. A similar procedure was done to the rest of the selected temperatures.

### 3.2. Oscillation Measurements

Viscoelasticity measurements were performed using oscillatory measurements through the sinusoidal strain technique. The oscillation experiments were measured employing a frequency from 0.1 rad/s to 100 rad/s in order to evaluate the *G′* (elastic modulus) and *G″* (viscous modulus). The complex viscosity is a measurement of the NADES viscoelasticity under the oscillatory frequency of w (rad/s).

The linear viscoelastic region (LVR) test was used to determine the critical strain for frequency sweep measurements. Both moduli were evaluated at a critical strain value of 0.1% according to the LVR test. Tables S2 and S3 summarized the flow curve and the pre-shear parameters used to run the experiment.

### 3.3. Thermogravimetric Analysis

The decomposition temperature, thermal stability, and onset temperature were measured on a PerkinElmer Pyris 6 TGA instrument. The NADES samples were left to equilibrate at 30 °C in order to measure the initial mass. The measurements were carried out at a temperature range of 30 to 700 °C using a constant rate of 5 °C min^−1^ under dry atmospheric conditions until the thermal degradation was complete. Periodic checks on the instrument were regularly done in order to verify the performance of all weight loss measurements using calcium oxalate.

### 3.4. Density

The density measurements of the NADES samples were carried out in the temperature range from 25 °C up to 85 °C und at atmospheric pressure conditions, using the DMA 4500 M densimeter provided by Anton Paar, which is based on the principle of the oscillating U-tube sensor. The uncertainty of the temperature is ±0.05 K, and the relative uncertainty of the density is ±0.00005 g·m^−3^. The calibration was conducted by referencing values of water densities [32].

### 3.5. Statistical Analysis of Experimental Data

Anton-RheoPlus Paar’s Rheoplus software was used to statistically interpret all of the experimental rheological data. The variations between the Bingham yield stresses for the NADES were evaluated using the statistical analysis of the Bingham model. For the association between the predicted and observable yield stress effects, the linear correlation coefficient (R^2^) was determined, indicating the importance of the Bingham model parameters. The findings of the rheological tests are repeatable, with an experimental error of less than 5%. All of the tests presented herein were tested at least twice.

## 4. Results and Discussion

### 4.1. Steady-State Flow

**Figure 1:**
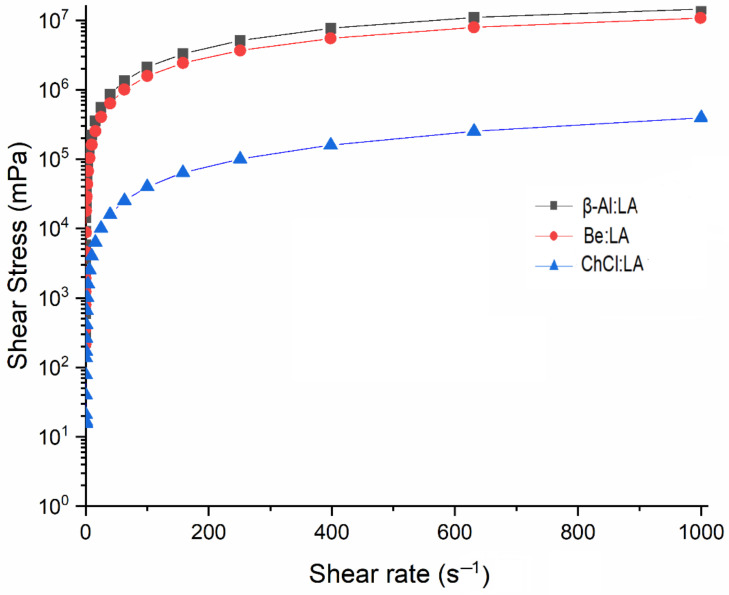
Shear stress variation as a function of applied shear rate for the studied three systems at 25 °C.

The distinct flow properties and rotary shear rest results of LA-based NADES have been shown considering time and temperature factors. The magnitudes of viscosity and the rheograms were determined utilizing rotational tests from 0.01 s^−1^ to 103 s^−1^ under similar sample conditions used in CO_2_ capture technologies [10]. Figure 1 shows a variation in the shear stress for every LA-based solvent with the shear rate. However, steadily elevated shear stress shows no interaction with the point of origin; therefore, the property of every sample is shown as a non-Newtonian characteristic at the conditions of room temperature [33]. The increase in the shear rate has also been found to be associated with the dominant effects of HBA, and the responses of NADES to the shear rate were considerably influenced by the characteristics of HBA. β-Al and Be showed the requirement of elevated yield stress as compared to ChCl, which is representative of the ability of NADES-related β-Al and Be linked to HBA to resist high yield stress as compared to the ability of ChCl. The shear stress progressively reached a steady state with the approach of the shear rate to 10^3^ s^−1^ showing that the solvents reached their viscosity limits. The shear-thinning properties were further shown by the damage to physical networks as a function of the shear rate. As a consequence, the NADES viscosity system’s nature roughly changed from non-Newtonian behavior to Newtonian behavior at higher shear rates [34].

Figure S1 represents ChCl:LA at four different temperature intervals (25 through 85 °C) with an increment of 25 °C under the effect of shear stress. Typical behavior of the shear-thinning effect was exhibited in all the flow curves of steady shear at all the predetermined intervals of temperature. As has been shown, the shear stress, depending on the shear rate, was reduced with the elevated temperature. The characteristic of this property was found to be similar to those seen in the previous studies, and it could be described by the breakdown of the structure due to structural thermal expansion [35]. On a further note, the research on ChCl:LA also showed that temperature affects the system while depending on the yield stress. In addition, the duration and slope of Hooke’s law are mainly related to the behavior of the ratio of HBA and HBD.

#### Thermo-Rheological Steady-Flow Behavior

**Figure 2:**
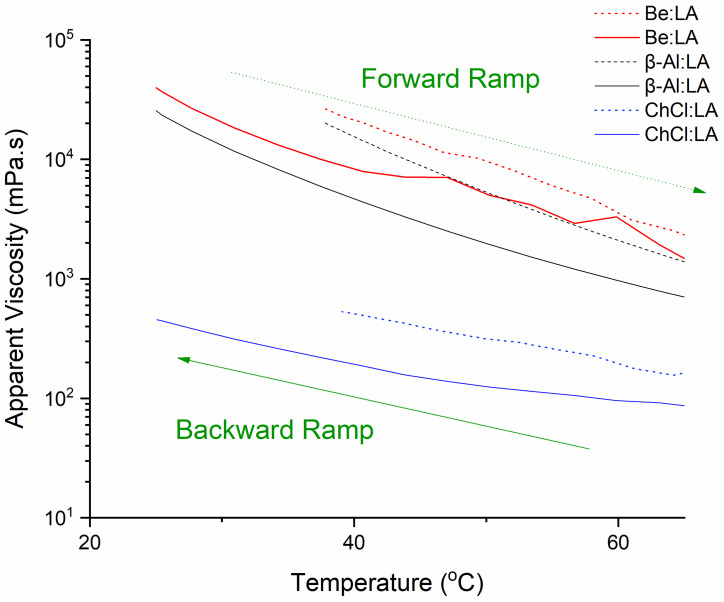
Effect of heating and cooling rate on the apparent viscosity at a low shear rate of 1 s^−1^. Curves are generated using 26 data points, temp ramp rate of 3 °C, and time ramp of 1.6 min.

The diverse range of temperature and conditions simulate production cycles and storage of the materials and determine the remote future and long-term usage of NADESs. Rheological testing can be used to predict the properties of the structure of the solvent, such as its resistance to temperature variations, under both cooling (representing backward ramp) and heating (representing forward ramp) conditions without huge costs on batch studies. As shown in Figure 2, approximately two decades of reduction can be found in the AV of β-Al:LA and Be:LA at the time of the heating process, whereas a comparatively mild level of reduction in ChCl:LA viscosity was found in the temperature ranging from 25 to 65 °C. Furthermore, the backward ramp (as shown by the reverse cooling process) from 65 to 25 °C represented almost similar profiles for the testing procedures. Eventually, this confirms that the LA HBD effect has a vital role in determining the shear flow.

**Figure 3:**
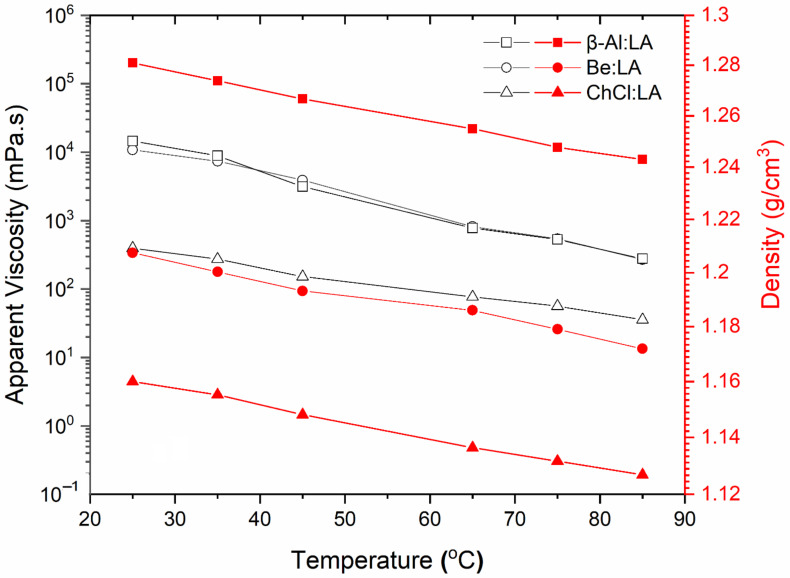
Effect of temperature on both the apparent viscosity (open symbols) and on density (filled symbols) at a shear rate of 1000 s^−1^ for the studied systems.

As illustrated by the LA-based NADESs in Figure 3, the AV of the systems steadily decreased with increasing temperature [9]. The viscosity of HBAs increased with the growth of the HBA hydrocarbon chain. Across the provided temperature range, both Be:LA and β-Al:LA systems showed almost the same viscosity-related characteristics. These systems were initially at about 2 × 10^4^ mPa·s at nearly 25 °C of temperature, i.e., room temperature, but then the viscosity gradually declined to a value about 66 times lower than the initial temperature and reached almost 300 mPa·s at the temperature of 85 °C. Whereas, the ChCl:LA system showed somewhat bulky and compact characteristics as compared to the other two systems, i.e., β-Al:LA and Be:LA, within the same temperature range. In the case of the ChCl:LA system, the viscosity declined by about ten times and reached a level of 40 mPa·s from 400 mPa·s with the increase in temperature. Apart from viscosity, NADES density demonstrated the same flow properties over a similar range of temperatures. For instance, the systems showed an almost linear monotonical decline in the density with increasing temperature. In the case of the density profiles of the systems shown in Figure 3, β-Al:LA demonstrated the highest level of density as compared to the other two systems. After looking at the density-related values of the systems, it can be found that β-Al:LA was denser than Be:LA, and Be:LA was denser than ChCl:LA at all studied temperatures. This property is a clear indication of the strong behavior of the hydrogen bonds that develop between HBA:HBD in the densest β-Al:LA system and the critical impact it has in the trend related to shear flow viscosity.

The three LA-based DES systems studied in this work were compared to density trends (Figure 3) obtained from previous studies for DES systems obtained by mixing ChCl as an HBD with non-natural organic acids such as levulinic acid [36] and phenylacetic acid [20] to investigate the effect of the type of HBA to HBD on the measured density. The density values obtained from natural organic HBA in this study, which were utilized to build DES systems, were more significant than those produced from non-natural organic acids, ranging from 1.15 to 1.10 g cm^−3^ with a temperature range of 20 °C to 90 °C [20]. Metallic salt-containing DES systems, on the other hand, had greater densities at room temperature, ranging from 1.3 to 1.6 g cm^−3^, which were higher than the organic and non-organic components in DES. Furthermore, across the temperature range of 20–90 °C, density values for the ChCl:levulinic acid DES system were found to be in the range of 1.14–1.10 g·cm^−3^ [36]. The LA-based DES revealed 1.28–1.24 g·cm^−3^, 1.21–1.17 g·cm^−3^, and 1.16–1.13 g·cm^−3^ for β-Al:LA, Be:LA, and ChCl:LA, respectively, in the study given here. The density of the ChCl:Urea DES system was found to be 1.25 g·cm^−3^ at 20 °C in research by García, et al. [37], which was in accordance with the predicted values of organic components. This indicates that the values of a comparable DES system made with ChCl and other organic-based components are similar.

**Figure 4:**
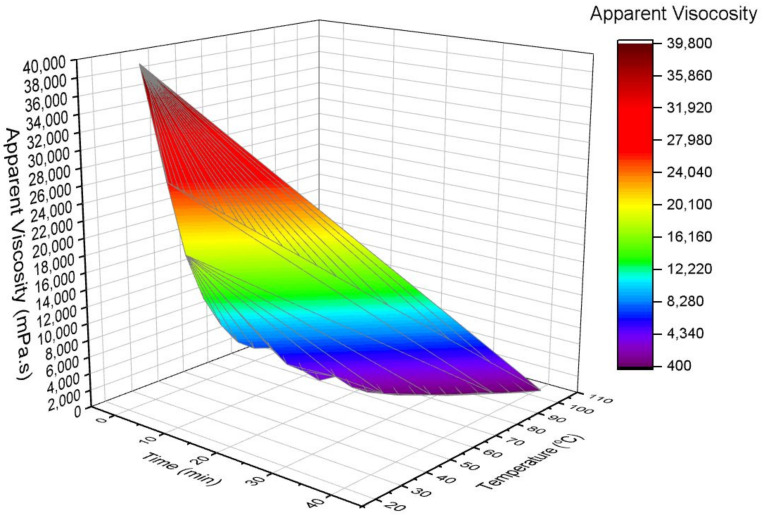
Surface 3D presentation of the effect of temperature and time on the apparent viscosity of Be:LA NADES system.

The rheology-related properties of NADES show the non-Newtonian characteristic because of the deterioration of the three-dimensional (3D) framework with the application of shear at an increased level of temperature and in a time-dependent manner. Figure 4 shows the 3D framework related to the influence of time and temperature on the AV of Be:LA system. It can also be found that time dependency affects Be:LA, and the viscosity reduces with the increase in temperature.

### 4.2. Oscillatory Tests

Oscillatory tests and measurements can be used to determine the elastic properties of the materials that have been developed recently. These measurements could help improve the knowledge related to the shear flow by increasing the level of understanding of the forces of interaction that appear between HBA and HBD of NADESs on a microscopic scale. Contrary to the findings of the shear flow measurements, oscillatory tests play a significant role in examining the molecular configurations of critical and sophisticated molecular interactions as they provide tests that can avoid damage to the networks of the materials, such as NADESs [38].

The storage modulus (*G′*, ratio of elastic stress over strain) and loss modulus (*G″*, ratio of viscous stress over strain) relating to the sum of energy stored and dissipated during deformation characterize the elastic properties of the materials. As seen in the following equation, the results of these two moduli are combined to form the complex modulus (G*).

(1) $$G^{*}=G^{\prime }+iG\prime \prime$$

where *i* is the unit imaginary number $\sqrt{-1}$. Furthermore, viscoelastic material acquires complex viscosity (η*), where ω is the angular frequency:

(2) $$\eta *=\frac{G^{*}}{\omega }$$

#### 4.2.1. Angular Frequency Sweep

Amplitude sweeping is the first step in oscillatory testing so that the linear viscoelastic region (LVR, as determined through the LVR test) of the sample can be defined. The LVR is considered a crucial point that can accurately determine the association of the molecular structure with the viscoelastic properties without any dependence on the enforced strain. The results were obtained over several oscillation frequencies ranging from 0.1–10^2^ rad/s under continual amplitude oscillation at a defined range of temperature, i.e., 25–85 °C.

**Figure 5:**
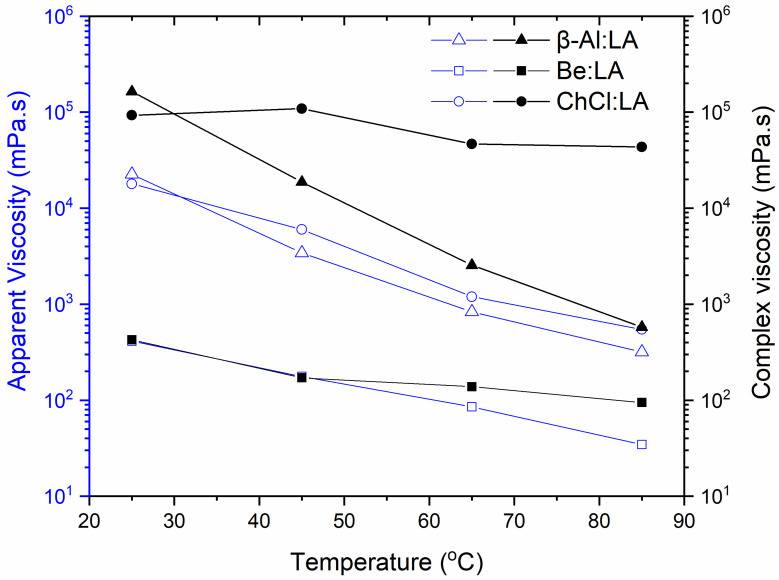
Effect of temperature on complex viscosity and apparent viscosity at an angular frequency of 1 rad·s*^−^*^1^ and a shear rate of 1 s*^−^*^1^ at temperatures from 25–85 °C.

Figure 5, as well as Figure S2, shows the complex viscous nature under the increased level of temperatures vs. the AV under two different cases. The first condition applied low applied frequency sweeping and a low shear rate, while the second case involved a relatively higher level of applied frequency sweeping and a shear rate under a regular strain at 0.1%. The selected strain value falls in-between the LVR range related to the NADES samples. The effect of the change in temperature on the complicated viscous nature of the samples was investigated at the temperature range of 25–85 °C and at a regular ω of 100 rad·s^−1^. The complicated viscous nature against temperature clearly indicated the temperature stability of the LA-based NADES over a range of temperatures. The Be:LA and β-Al:LA NADES demonstrated a steep decline in viscosity with temperature, whereas the viscosity of ChCl:LA declined in a uniform manner. The comparison of the complex viscous nature of the three systems showed that the ChCl:LA system was able to establish a high level of resistance to temperature change in relation to viscosity. It showed a gel-like structural property, whereas the other two systems showed a steep decline representing the deterioration of the gel-like structure with temperature. It is important to note that the behavior in complex viscosity determination was similar to the apparent shear viscosity determined in shear-flow characteristics for all the studied NADES. Eventually, the ChCl:LA system showed increased resistance to change in structure, and accumulation of the network was found at increased temperatures. This indicates the most resistive molecular structure and behavior of network building under high temperatures for ChCl:LA.

#### 4.2.2. Dynamic Temperature Ramp Sweep

**Figure 6:**
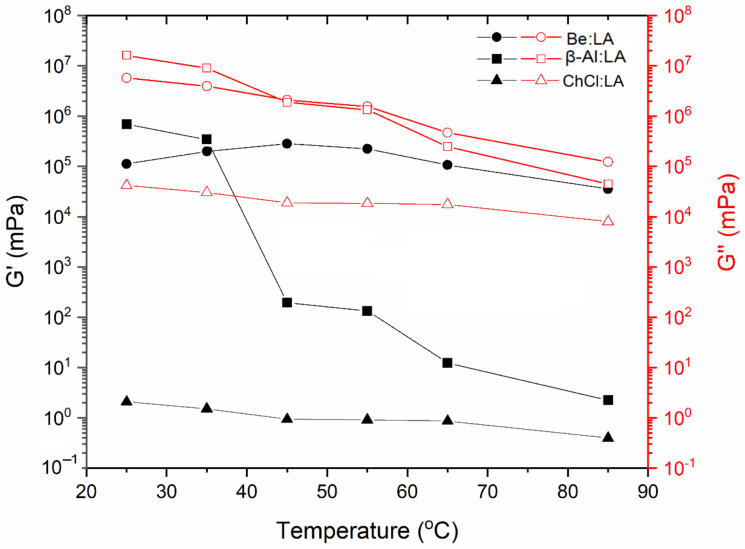
Effect of temperature on elastic modulus (*G′*) and viscous modulus (*G″*) at a high angular frequency of 100 rad·s^−1^ at temperatures from 25–85 °C.

**Figure 7:**
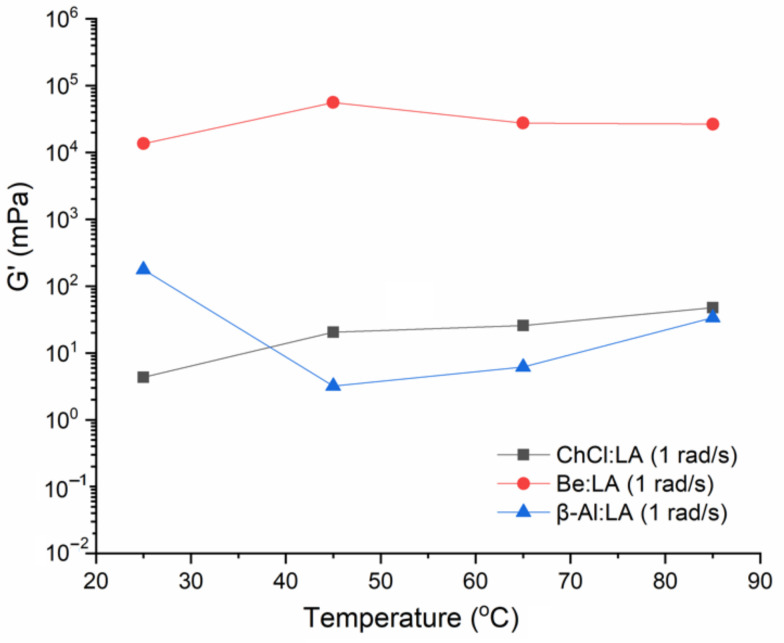
Elastic modulus (*G′*) at the low angular frequency at temperatures from 25–85 °C.

**Figure 8:**
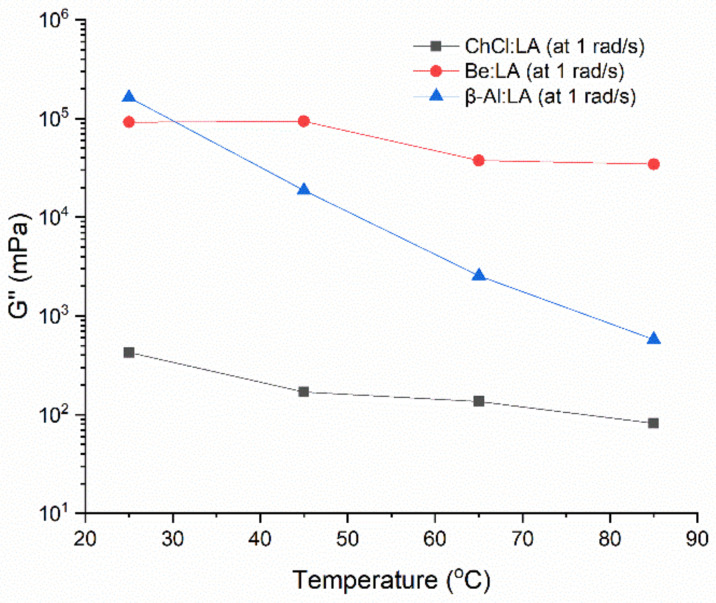
Viscous modulus (G″) at the low angular frequency at temperatures from 25–85 °C.

Figure 6, Figure 7 and Figure 8 represent the *G′* and *G″* for the three systems as a function of temperature in the range from 25–85 °C at a regular higher ω of 100 rad·s^−1^. The two systems, including ChCl:LA and Be:LA, show consistent and stable properties over the applied range of temperatures. A slight alteration in the flow characteristics was found with the increase in temperature for the *G′* and *G″*, whereas sharp damage to the structure in *G′* was found for β-Al:LA after the temperature of 35 °C, which is representative of the good physical stability as the internal structure could not be easily changed. On a further note, the viscoelastic properties of the LA-based NADESs demonstrated that *G″* is better than *G′*. This showed that viscosity and low stiffness of the samples of NADESs was retained over the solid-like elastic properties. Therefore, the samples were mostly viscous liquid, and the samples rarely had a solid nature [30]. The dissimilarities between *G′* and *G″* show a gel-like material by the existence of the flow components in this structure, and as *G′* begins to diminish (i.e., as *G′* approached zero), an ideal type of viscous flow property with a decreased level of rigidity was found. The larger values of the *G″* and *G′* in the systems, including Be:LA and β-Al:LA NADES, can be considered as their representation of natural structural strength and rigidity at the gel-like state, while the ChCl:LA system has a softer viscoelastic structure. These remarkable properties of different NADESs over a range of temperatures make them suitable agents for industrial operations.

Figure S3a,b represent the dynamic test finding of the frequency sweep measurement. The test was done by examining the *G′*, *G″* vs. ω for the LA-based NADES systems from room temperature to 85 °C. Under the conditions of room temperature, all the systems represented higher *G″*as compared to *G′*. This indicates that every LA-based NADES system has a general behavior of a liquid, and almost the same structural behavior was represented by the LA-based NADES systems at 85 °C.

Figure S3c,d demonstrate the longest linear viscoelastic area of *G′* for Be:LA. This structure of the Be:LA initially represented a semi-solid framework showing a constant straight trend without being dependent on ω. The breakdown and deviation from G′ constant behavior happened for ChCl:LA and β-Al:LA at various levels of ω with the temperature elevation. The change from the original structural behavior at low levels of ω was found to be higher with the increase in temperatures. This indicates that a decreased level of stress is necessitated for disturbing the structures at reduced temperatures compared with the increased temperatures for easy damage to the structure. On a further note, a structural reestablishment was found at every temperature for NADESs. The structure recovery leads to the initial structure at higher ω and room temperature, and a steady recovery was found at other temperatures. Eventually, it can be said that this characteristic of LA-based NADESs shows that at isothermal conditions, the typical correlation of NADES at different temperatures is in agreement with those analogies at individual temperature measurements.

### 4.3. Thermal Stability

Figure S4 represents the thermogravimetric analysis (TGA) conducted on the three systems to determine thermal stability. Both Be:LA and β-Al:LA systems presented almost the same trends. The systems showed a steady singular-step decomposition with an estimated thermal decomposition temperature (T_dec_) of about 220 °C. The ChCl:LA NADES system decomposition pattern also showed a single-step deterioration with an estimated T_dec_ of about 187 °C. The results are an obvious representation of the effect of HBA present and have a critical role in showing the thermal stability of the NADES systems. On a further note, the entire observation is also representative of their probable application as pre-combustion as well as post-combustion CO_2_-capturing entities [30,35,39].

## 5. Mathematical Modeling

The behavior of the fluids could be interpreted with the help of viscosity and flow curves and with the notice of yield stress and AV. The Bingham model is among the commonly used mathematical models related to fluids showing non-Newtonian characteristics. This model has been used for the assessment and transformation of rheogram data collected from experimental findings into an interpretable rheological property of several LA-based NADES systems.

The Bingham equation, associated with the Bingham model, represents two important parameters related to the rheological model. It is employed for the illustration of the viscosity function and for the estimation of the values of the dynamic yield stress of the fluids showing the property of a rigid body at a decreased level of stress while showing the viscous nature at an increased level of stress [35,40]. Moreover, the Bingham model considers fluids having a shear-thinning property at decreased levels of shear rates. Furthermore, the model also links to the areas with a constant slope and encompasses elevated shear rates [41]. It is also important to note that the model also deals with the fluids showing the property of Bingham plastic (such as toothpaste), the AV of which seems infinite until a critical shear stress value is reached [33]. The Bingham equation has been shown in Equation (2).

(3) $$\tau \;={\;\tau }_{o}+{\;\eta }_{o}\gamma$$

where${\;\tau }_{o}$ is the Bingham yield stress, τ represents the shear stress, γ is the shear rate, and$\;\eta_{o}$ is the Bingham plastic viscosity. The $\tau_{o}$ indicates the minimum stress required to disrupt the network structure that would initiate the flow. The $\tau_{o}$ magnitude significantly describes the mixture’s behavior; hence, a value below${\;\tau }_{o}$ is considered as a rigid solid and vice versa [35].

The yield stress of all the systems (as presented in Table 3) represented a reduction with the elevated temperature. The differences in the yield stress could be related to the alterations in the mobility of the molecules, for example, the rigidity of the chain and forces of interaction that are developed between HBD and HBA of the NADESs and the molecular weight of the systems [19]. The variation in the actual yield stress magnitudes was almost the same as the data related to viscosity [35]. The yield stress (τ_o_) of all three systems represented higher magnitudes for Be:LA and β-Al:LA (i.e., 233,000 and 338,000 mPa, respectively), showing their intensive requirements for energy to disrupt the network structure for initiating the flow property, whereas the low values of τ_o_ in the case of the ChCl:LA system show benefits for industrial application such as CO_2_-related processes, particularly during pumping. Regarding the ChCl:LA system, the reported values were fluctuating, though they were below the acceptable limits of error. The statistical values of the viscosity-related data were fitted, and a R^2^ showed that the Bingham regression completely fits the observed data values, as R^2^ is approximately equal to one for every system.

## 6. Conclusions

The research on stationary shear rheometry was conducted to describe the rheology-related properties of three different LA-based NADES systems with a predetermined 1:1 mole-to-mole ratio at oscillatory and rotational shear, representing the effect of temperature in field operations on flow properties and time-dependent properties. The LA-based NADES systems presented in this study showed the characteristics of a non-Newtonian fluid depending on time and their shear-thinning properties. Rheological studies also demonstrated the dissimilarity in the strength and viscosity of the viscoelastic behavior of the investigated systems. The AV influenced by temperature demonstrated potent shear-thinning effects similar to the alternative IL sorbents [9]. The viscoelastic measurements in the form of the dynamic frequency sweep tests aided in concluding that the ChCl:LA has a higher potential in the process of CO_2_ capture as compared to the systems of Be:LA and β-Al:LA, as it was found to have a more resistive and compact nature showing an accumulating behavior at higher temperatures. The thermal stability could also enhance the thermo-physico rheological characterization by emphasizing the influence of the nature of HBA. The τ_o_ property reported from the Bingham mathematical model established that the requirement of stress is considerably less for ChCl:LA in comparison to the extreme requirements of Be:LA and β-Al:LA. Although much work has been devoted to exploring NADES’ uses in many industrial applications, NADES’ contribution to rheological characterization remains restricted. Studies of rheology will not only help determine the physical or chemical structure of the NADES, but they will also determine if materials are good enough to be made and used on a larger scale in industry.

## Figures and Tables

**Table 1:** Chemicals used in the preparation of the NADES systems.

SN	Chemical	Chemical Structure	HBA/HBD	Remark at Room Temperature
1	Choline chloride	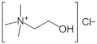	HBA	Solid powder
2	Betaine	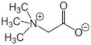	HBA	Solid Powder
3	β-alanine	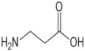	HBA	Solid Powder
4	Lactic acid	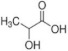	HBD	Viscous liquid

**Table 2:** List of LA-based NADES samples and their Karl Fischer titration results.

SN	NADES	Abbreviation	Molar Ratio	Water Content (ppm)	Water Content (%)
1	Choline chloride:lactic acid	ChCl:LA	1:1	27,787	2.8
2	Betaine:lactic acid	Be:LA	1:1	7631	0.7
3	β-alanine:lactic acid	β-Al:LA	1:1	44,417	4.4

**Table 3:** Bingham yield stress and plastic viscosity data for the studied systems at temperatures from 25–85 °C.

	Temperature (°C)	τ_o_ (mPa)	ɳ_o_ (mPa·s)	R^2^
β-Al:LA	25	338,000	15,600	0.9811
	45	11,214	3164.5	0.9995
	65	578.48	783.23	1.0000
	85	222.14	279.13	1.0000
Be:LA	25	233,000	11,300	0.9860
	45	27,389	3975.9	0.9985
	65	2656.5	818.19	0.9999
	85	974.50	268.56	1.0000
ChCl:LA	25	0.0181	0.4003	1.0000
	45	0.0056	0.1556	1.0000
	65	0.0244	0.0790	1.0000
	85	0.0153	0.0393	1.0000

## Data Availability

Not applicable.

## Supplementary Materials

The following supporting information can be downloaded at: https://www.mdpi.com/article/10.3390/ma15114027/s1, Figure S1: The variation of shear stress as a function of applied shear rate for ChCl:LA NADES systems at different temperatures from 25–85 °C; Figure S2: Complex viscosity & apparent viscosity variation for Be, B-Al, ChCl:LA NADES systems at high angular frequency (100 rad. s^−1^) and high shear rate (1000 s^−1^) under heating from ambient temperature conditions to 85oC; Figure S3a: The elastic modulus (G′) at an angular frequency range from 0.1–100 rad. s^−1^ for Be, B-Al, and ChCl:LA at room temperature conditions; Figure S3b: The viscous modulus (G′′) at an angular frequency range from 0.1–100 rad. s^−1^ for Be, B-Al, and ChCl:LA at room temperature conditions; Figure S3c: The elastic modulus (G′) at an angular frequency range from 0.1–100 rad. s^−1^ for Be, B-Al, and ChCl:LA at high temperature; Figure S3d: The viscous modulus (G′′) at an angular frequency range from 0.1–100 rad. s^−1^ for Be, B-Al, and ChCl:LA at high temperature; Figure S4: TGA thermographs of LA-based NADES systems; Table S1: Shear flow parameters; Table S2: Pre-Shear flow parameters Table S3: Oscillatory shear flow parameters.

## Nomenclature

β-Al	β-Alanine
AV	Apparent Viscosity
Be	Betaine
ChCl	Choline Chloride
HBAs	Hydrogen Bond Acceptors
HBD	Hydrogen Bond Donor
ILs	Ionic Liquids
LA	Lactic Acid
LVR	Linear Viscoelastic Region
NADES	Natural Deep Eutectic Solvents
TGA	Thermogravimetry Analysis
