# Supplementary material for: An Experimental Investigation on the Thermo-Rheological Behaviors of Lactic Acid-Based Natural Deep Eutectic Solvents
Source: Materials (Basel). 2022 Jun 6;15(11):4027. doi: 10.3390/ma15114027 (PMC9182069; doi:10.3390/ma15114027)
Supplement: Supplementary file 1 [file materials-15-04027-s001.zip › materials-1672847-supplementary.pdf]

An experimental investigation on the thermo-rheological behaviors of lactic acid based natural deep eutectic solvents

Yousef Elhamarnah [b], Wadha Almarri [b], Asma Albadr [b], Alanoud Almalki [b], Mashael AlRasheedi [b], Nora Mohamed [b], Izzah Fatima [b], Mustafa Nasser [ab], Hazim Qiblawey [b]*

[a] Gas Processing Center, College of Engineering, Qatar University, Doha, Qatar
[b] Department of Chemical Engineering, College of Engineering, Qatar University, Doha, Qatar

Corresponding Author: Mustafa Nasser m.nasser@qu.edu.qa

Supplementary information

Table S1: Shear flow parameters

Parameter	value
Shear rate (1/s)	0.01-1000
Data points	50
Single point duration (s)	12
Temperature range (^{o}C)	25, 45,65,85,105

Table S2: Pre-Shear flow parameters

Parameter	value
Shear rate (1/s)	0.01 (constant)
Data points rate (point/s)	5

Table S3: Oscillatory shear flow parameters

Parameter	value
Shear strain (%)	10 (oscillating)
Data points rate (Point/s)	25
Initial angular frequency (rad/s)	100
Final angular frequency (rad/s)	0.1

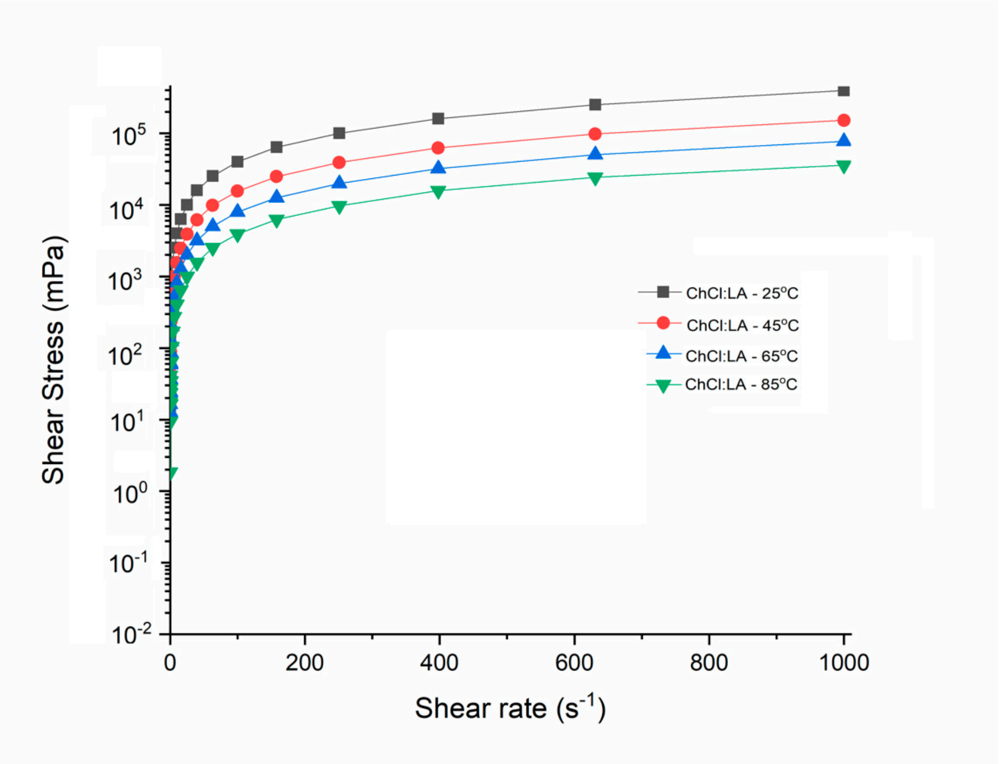

Figure S1. The variation of shear stress as a function of applied shear rate for ChCl:LA NADES systems at different temperatures from 25-85 °C

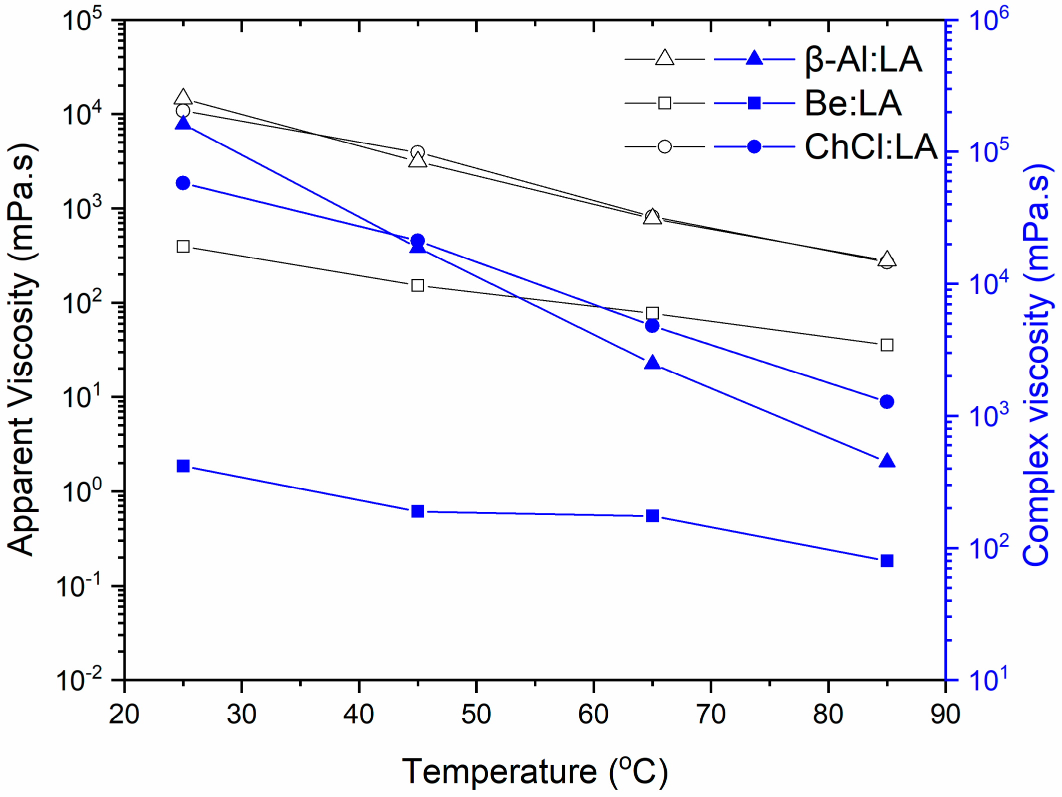

Figure S2. Complex viscosity & apparent viscosity variation for Be, B-Al, ChCl:LA NADES systems at high angular frequency (100 rad. s^{-1}) and high shear rate (1000 s^{-1}) under heating from ambient temperature conditions to 85°C

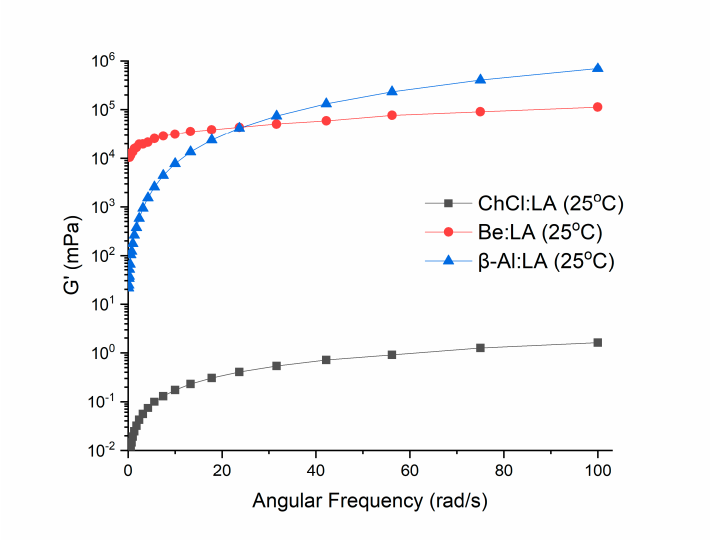

a

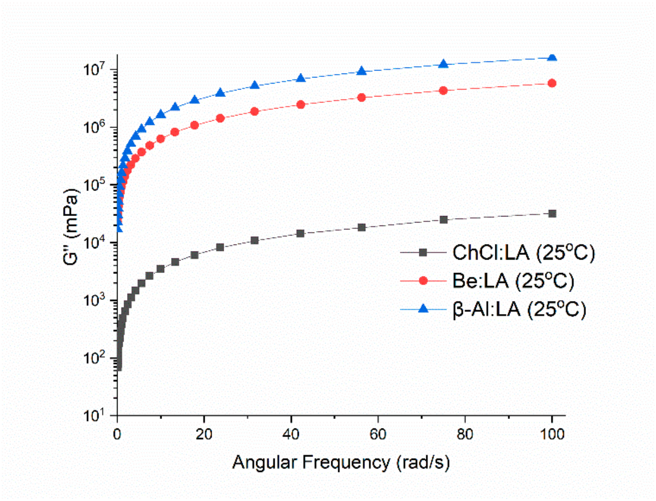

b

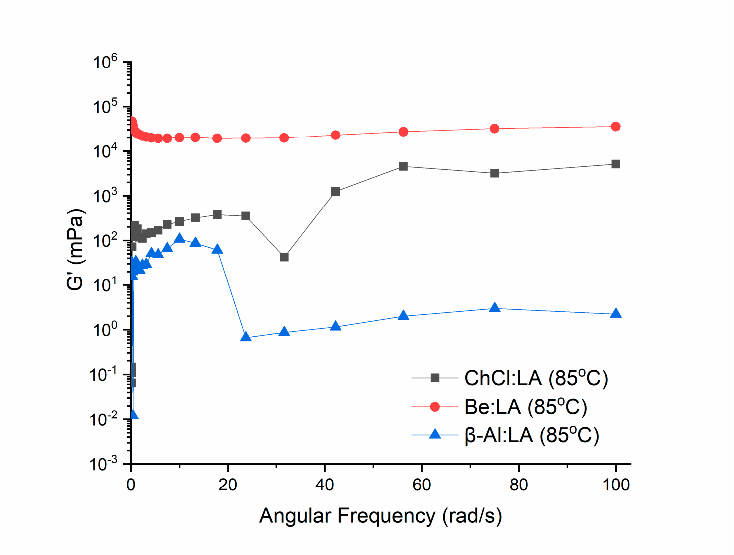

c

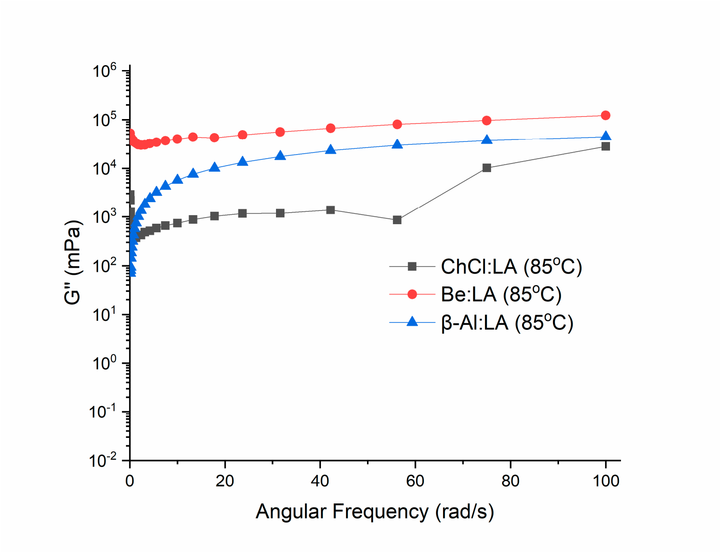

d

Figure S3. (**a**) The elastic modulus (G′) at an angular frequency range from 0.1–100 rad. s^{-1} for Be, B-Al, and ChCl:LA at room temperature conditions. (**b**) The viscous modulus (G′′) at an angular frequency range from 0.1–100 rad. s^{-1} for Be, B-Al, and ChCl:LA at room temperature conditions. (**c**) The elastic modulus (G′) at an angular

frequency range from 0.1–100 rad. s^{-1} for Be, B-Al, and ChCl:LA at high temperature. (**d**) The viscous modulus (G″) at an angular frequency range from 0.1–100 rad. s^{-1} for Be, B-Al, and ChCl:LA at high temperature.

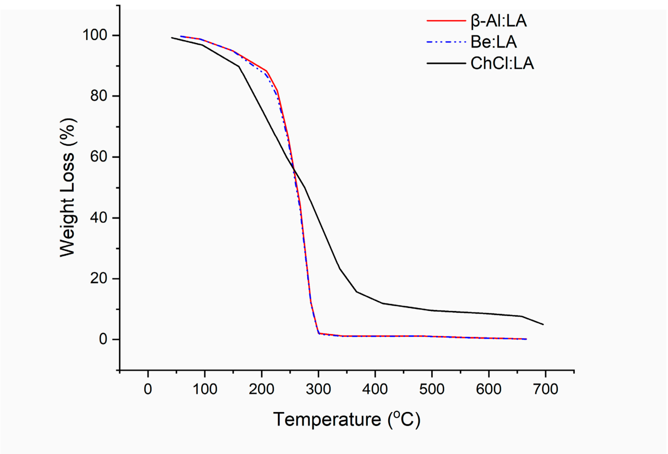

Figure S4. TGA thermographs of LA-based NADES systems.
